# Chrysin Attenuates Gentamicin-Induced Renal Injury in Rats Through Modulation of Oxidative Damage and Inflammation via Regulation of Nrf2/AKT and NF-kB/KIM-1 Pathways

**DOI:** 10.3390/biomedicines13020271

**Published:** 2025-01-23

**Authors:** Talat A. Albukhari, Rehab M. Bagadood, Bayan T. Bokhari, Waheed A. Filimban, Hatem Sembawa, Nani Nasreldin, Hossam E. Gadalla, Mohamed E. El-Boshy

**Affiliations:** 1 Department of Hematology and Immunology, Faculty of Medicine, Umm Alqura University, Makkah 24382, Saudi Arabia; 2 Department of Clinical Laboratory Sciences, Faculty of Applied Medical Sciences, Umm Al-Qura University, Makkah 24382, Saudi Arabia; rmbagadood@uqu.edu.sa (R.M.B.); btbokhari@uqu.edu.sa (B.T.B.); 3 Pathology Department, Faculty of Medicine, Umm Alqura University, Makkah 24382, Saudi Arabia; wahfilim@gmail.com; 4 Department of Surgery, Faculty of Medicine, Umm Alqura University, Makkah 24382, Saudi Arabia; hasembawa@uqu.edu.sa; 5 Department of Pathology and Clinical Pathology, Faculty of Veterinary Medicine, New Valley University, El-Kharga P.O. Box 72511, Egypt; nani_nasreldin@vet.nvu.edu.eg; 6 Department of Clinical Pathology, Faculty of Veterinary Medicine, Mansoura University, Mansoura P.O. Box 35516, Egypt; gadallahha@gmail.com

**Keywords:** chrysin, gentamicin, nephrotoxicity, antioxidants, proinflammatory cytokines, inflammatory transcription factors

## Abstract

Background: Gentamicin (GM) is extensively used as an antibiotic for the treatment of infections caused by Gram-negative bacteria. Oxidative stress and proinflammatory cytokines are implicated in GM-induced renal damage. Chrysin (CH), also known as 5,7-dihydroxyflavone, has been used in traditional medicine to treat various kidney disorders. The aim of this study was to investigate the antioxidant, anti-apoptotic, and anti-inflammatory effects of CH against nephrotoxicity induced by GM. Methods: Male rats were separated into four equal groups: a negative control group (NC), a CH-treated group (100 mg/kg/day per os), a group treated with GM (100 mg/kg/day IM), and a group treated with both GM and CH (100 mg/kg/day), for 10 days. Blood and urine renal markers were investigated. Results: GM caused increases in the serum creatinine and urea levels and decreases in creatinine clearance, urine flow, and urine volume in the GM-treated rats. Moreover, there were increases in the levels of IL-1β, TNF-α, IL-18, and MDA in the renal tissues, with an augmented expression of NF-κB/KIM-1, as well as decreases in antioxidant marker (GSH, GPx, CAT, and SOD) activities and decreased expressions of the anti-inflammatory transcription factors Nrf2 and AKT. The simultaneous treatment with CH in the GM-treated group protected renal tissues against the nephrotoxicity induced by GM, as demonstrated by the normalization of renal markers and improvement in histopathological damage. Conclusions: This study reveals that CH may attenuate GM-induced renal toxicity in rats.

## 1. Introduction

Gentamicin (GM) is the oldest aminoglycoside antibiotic, commonly used to treat renal illness and intense infections caused by Gram-negative bacteria. Nevertheless, GM produces significant ototoxicity and is harmful to the renal system, which restricts its continued administration [1]. In addition, GM exhibits dose-related nephrotoxicity and accounts for 20% of all cases of acute kidney failure [2]. The types of kidney damage associated with GM therapy include renal tubular necrosis, tubular edema, glomerular damage, renal apoptosis, and changes in kidney function markers. GM induces kidney damage by selectively accumulating in the epithelial cells of proximal convoluted tubules in the renal tissues [3]. Reactive oxygen species are produced and renal antioxidant enzyme activities are inhibited when GM accumulates in renal tubular cells [4]. GM nephrotoxicity is related to the occurrence of renal oxidative stress, which plays an important role in increasing the proinflammatory cytokines that cause renal apoptosis and impair various renal functions [5]. The fundamental pathways of GM-caused renal damage are the enhancement of renal oxidative stress, the production of reactive oxygen species such as malonaldehyde, the upregulation of proinflammatory molecules (IL1β, IL6, IL18, and TNF-α), and a reduction in the antioxidant defense system, including decreased glutathione (GSH), glutathione reductase (GR) [1,6], catalase (CAT), and superoxide dismutase (SOD) [7,8,9]. The molecular pathogenesis of GM-induced nephrotoxicity has been linked to an increased activation of pathological transcription factors, such as kidney injury molecules (KIMs) [10,11] and nuclear factor kappa B (NF-κβ) [12,13]. Additionally, GM is known to inhibit anti-inflammatory transcription molecules, such as nuclear factor–erythroid 2-related factor-2 (Nrf-2), AKT [14], and IL10 [8,15]. To date, GM has remained an important antibiotic in the treatment of numerous diseases and life-threatening illnesses triggered by different Gram-negative bacterial infections [16].

Chrysin is a flavonoid compound (5,7-di-OH-flavone) found in honey, propolis, blue and trumpet flowers, fruits, vegetables, mushrooms, and various medicinal plants. It is classified in the flavone class of polyphenolic complexes and consists of 15 carbon atoms, a unique fused two-ring structure, two hydroxyl groups, and a phenyl ring [17]. CH has unique properties due to the absence of oxygenation in its structure rings, which enhances its bioavailability, augments its protein strength, and prevents its proteolytic degradation. It also has potent antioxidant and anti-inflammatory effects [18]. CH is derived from the essential amino acids phenylalanine and tyrosine, which have significant dietary value, economic importance, and phytomedicinal therapeutic value [19]. It is a potent free radical scavenger that donates a hydrogen atom via its phenyl ring and -OH groups [20]. CH has many pharmacological activities, including a protective effect against damage to multiple organs, such as the liver, kidney, CNS, and heart, and it inhibits atherogenesis, viral activity, allergy, and hyperlipidemia [19,21,22]. CH has been proven to prevent kidney damage caused by renal ischemia, carbon tetrachloride, 5-Fluorouracil, and paclitaxel-induced nephrotoxicity by inhibiting oxidative stress, reducing the levels of proinflammatory molecules (such as IL1β, IL6, and TNF-α), and lowering the apoptosis index [23,24,25,26]. CH also attenuates renal carcinogenesis through its antioxidant and anti-inflammatory effects by suppressing transcription factors that play a critical role in pathogenesis (such as NF-κβ) [17]. Moreover, CH has been documented to provide renal protection against sodium arsenate, lead acetate, colistin, and cisplatin by inhibiting proinflammatory cytokine markers and oxidative stress while enhancing antioxidant markers such as GSH, GR, GPx, CAT, and SOD [27,28,29]. In addition, CH improves renal markers and ameliorates renal fibrosis induced with cyclosporine by inhibiting TGF-β_1_ [30]. There is an ongoing search for natural products that offer nephroprotection effects to counteract kidney damage. Treatment with natural antioxidants may help protect the kidney from GM-induced renal damage. However, no studies in the literature have examined the renal defensive impact of CH in the context of GM toxicity. Therefore, the present study was designed to investigate the molecular protective mechanisms of CH, along with its antioxidant and anti-inflammatory effects on GM-induced renal damage.

## 2. Materials and Methods

### 2.1. Chemical Reagents

Gentamicin sulfate is an aminoglycoside composed of six carbon rings with the molecular formula C21H43N5O7; it was purchased from Schering Pharmaceutical Co. (Kenilworth, NJ, USA). Chrysin, also known as 5,7- di-OH-flavone, has the chemical formula C15 H10 O4, and it was acquired from SIGMA (Aldrich, CO., Burlington, MA, USA) with a purity level of 97% (Cat# C80105). Chrysin was dissolved in 0.5% methylcellulose [31].

### 2.2. Experimental Design

Thirty-two male albino rats weighing 180 ± 10 g were acquired and housed in standard environments following ethical guidelines (temperature of 24 ± 2 °C and 12:12 h light/dark cycle). The experimental animals were provided a basic ration and had access to water ad libitum during the study period. This study followed the guidelines of the Declaration of Helsinki and the World Medical Association. Each rat was kept in a separate plastic cage. After a week of acclimation, the rats were separated into 4 equal groups: a negative control (NC) group treated with normal saline via intraperitoneal injection, a group treated with CH per os at 100 mg/kg/day for 10 concussive days, a positive control group treated with GM via intraperitoneal injection at a dose of 100 mg/Kg/day for 10 consecutive days, and a final group that received both GM and CH for 10 consecutive days (GM + CH). The approved treatment protocols for CH and GM followed the protocols described in [28,32], respectively.

### 2.3. Types of Samples Collected

At the end of the 10-day period, each rat was kept in a metabolic cage, and 24 h urine specimens were collected. The rats were sedated with ketamine (100 mg/kg) and xylazine (10 mg/kg) via intraperitoneal injection. Blood specimens were collected from the retro-orbital plexus of the rats and centrifuged, and the serum was kept at −20 °C. The kidneys were removed from the experimental rats, and a portion was fixed in 10% formalin for histopathology. To measure renal antioxidants and cytokines, total protein was extracted from renal samples (400 mg) using the RIPA lysis buffer including protease inhibitors, and the final volume was diluted to 1000 µg/mL after being assessed with a BCA protein assay (MERCK, Millipore, Burlington, MA, USA). Total RNA was obtained from the residual renal samples using a high-capacity reverse transcription kit and was then utilized for CDNA production (Creative, Biogen, Shirley, NY, USA).

### 2.4. Assay of Renal Function Markers

Blood levels of creatinine (Cr), urea, total protein, albumin, and 24 h U Cr with urine protein were estimated using the kits supplied by Human Diagnostic Co., Wiesbaden, Germany, according to the manufacturer’s instructions. All these tests were assayed using a completely automated analyzer (Wiener Lab, Carouge, Switzerland). The 24 h U level and Cr clearance (Cr-Cl) were determined as described in [33].

### 2.5. Assay of Proinflammatory, Antioxidant, and Oxidative Markers 

The renal proinflammatory cytokines—TNF-α, IL1β, IL6, IL10, and IL18—were estimated using commercially available ELISA kits from MyBioSource (San Diego, CA, USA—Abcam-Cambridge, UK). In addition, the levels of the renal antioxidant markers— GSH, SOD1, CAT, GPx1, MDA, protein carbonyl, and H2O2 (Cayman Co., Ann Arbor, MI, USA)—as well as the levels of the damage molecules—HSP25, Caspase-3, and BCL-2—were assayed using commercially available ready-to-use kits from CUSABIO (Houston, TX, USA). All the investigated parameters were quantified in replicated samples using a computerized ELISA device (Poweam Medical, CO., Nanjing, China), following the manufacturer’s protocols.

### 2.6. Quantitative qRT-PCR of Pathogenic Molecules

The whole-kidney mRNA levels of AKT, Nrf2, NF-κβ, KIM-1, Caspase-3, and BCL-2 transcription factors in the different experimental groups were evaluated via quantitative RT-PCR. In brief, total RNA was obtained using TRIzol. The cDNA was utilized as a template in the PCR, and the mRNA expression of the target transcription factors was assessed. An amount of 1 µg of total RNA was used to synthesize the cDNA using a cDNA production kit (Bio-Rad, Hercules, CA, USA), together with the SYBR Green master mix; the various primers used are displayed in Table 1. The mRNA level of actin was employed as the internal control, according to the manufacturer’s instruction. The preconditions were 30 cycles at 94 °C for 30 s, 56 °C for 30 s, and 72 °C for 25 s. The changes in expression were computed using the 2^−ΔΔCt^ method.

### 2.7. Histopathology Study

Specimens were collected from the renal tissues of each rat and treated using the traditional method of fixing in methanol and then embedding in paraffin. Sections of 5 μm in thickness were sliced from individual tissue blocks and stained with Hematoxylin and Eosin (Abcam, Waltham, MA, USA), following the standard histological protocols.

### 2.8. Histopathological Semi-Quantitative Analysis

The renal sections were inspected semi-quantitatively in 3 fields of view at an amplification of 100×. The guide for the histopathological scoring of the main lesions is shown in Table 2. The score of each lesion was established for each field (none: 0; mild: 1; moderate: 2; severe: 3). The mean of all scores was calculated for each rat.

### 2.9. Statistical Analysis

The data were analyzed using SPSS version 21, and the Kolmogorov–Smirnov test and Levene’s test were conducted to check data normality and homogeneity, respectively. The non-parametric Kruskal–Wallis test was used to analyze the histopathological scoring data across the treatment groups, with the Mann–Whitney U test employed for two-group comparisons. The data are presented as the mean ± SD of three replicates. One-way ANOVA followed by post hoc Tukey’s test was used to compare the treatment groups. A *p*-value  <  0.05 was considered statistically significant.

## 3. Results

### 3.1. Serum and Urine Kidney Function Tests

The GM group presented a significant increase in serum Cr and urea, together with evident proteinuria and oliguria (*p* < 0.01 for all), showing a significant reduction in serum protein, albumin, and 24 h U Cr clearance (*p* < 0.01) compared to the NC rats. The rats treated with both CH and GM demonstrated marked decreases in blood Cr and urea, together with increased 24 h U Cr concentrations and Cr-Cl, when compared to the GM group, as shown in Table 3. However, the levels of the serum and renal indicators in the CH + GM group were not significantly different from those of the NC group. Additionally, the levels of all serum and urine kidney markers were comparable between the CH and NC groups (Table 3).

### 3.2. Concentrations of Inflammatory and Antioxidant Markers in Kidney Tissues

The renal concentrations of TNF-α, IL1β, IL6, IL18, HSP25, MDA, H_2_O_2_, and protein carbonyl were increased significantly in the GM group, whereas those of IL10, GSH, SOD1, GPx1, and CAT were reduced, compared to the NC rats (Table 4). The levels of proinflammatory and oxidative markers decreased significantly in the kidney tissue samples from the CH + GM-treated rats compared to the GM-treated group, while the level of the anti-inflammatory mediator IL10 and those of antioxidant markers increased significantly (Table 4). Furthermore, the renal concentrations of IL10, HSP25, GSH, SOD1, GPx1, CAT, protein carbonyl, and MDA in the CH + GM group were comparable to those of the NC group (Table 3). The levels of the renal apoptosis markers Caspase-3 (7.57 ± 1.16 ng/mL) and BCL-2 (1.25 ± 0.71 ng/mL) in the GM group were comparable to those of the NC group (1.36 ± 0.36 and 2.53 ± 0.63 ng/mL, respectively), but a marked reduction was observed in the CH + GM-treated group (4.23 ± 0.64 and 1.70 ± 0.65 ng/mL, respectively) compared to the GM group.

### 3.3. Expression of Markers Indicative of Kidney Pathogenesis

**Figure 1:**
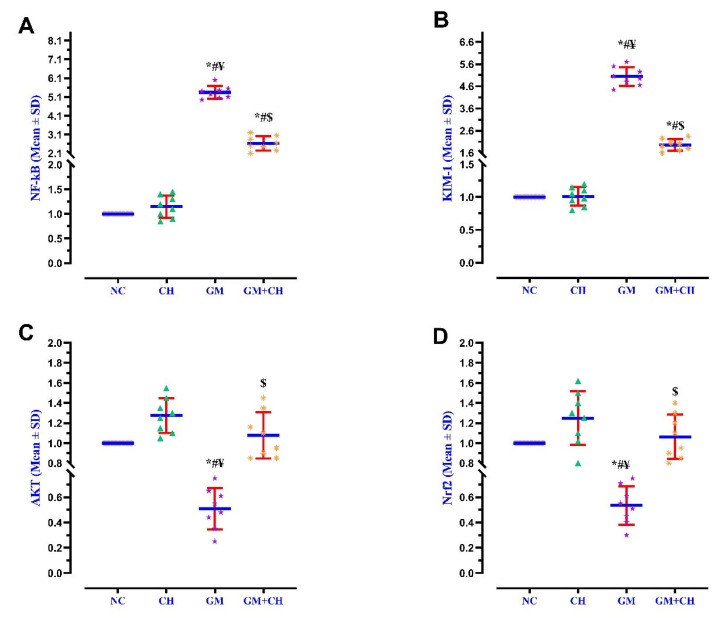
Interleaved scatter plots presenting the mean ± SD of the relative expression of NF-kB (**A**), KIM-1 (**B**), AKT (**C**), and Nrf2 (**D**) in the renal tissues obtained from the GM and GM + CH groups compared with their relative expression in the NC and CH groups. Dots, triangles, stars, and asterisks indicate the values of NF-kB, KIM-1, AKT, and Nrf2 for the rats in each group (*n* = 8). Red error bars represent the ± standard deviation of the mean, and the blue lines indicate the mean values of the relative expression of these genes in each group. Statistical significance is indicated as follows: * = *p* < 0.05 compared with the NC group; ^#^ = *p* < 0.05 compared with the CH group; ^$^ = *p* < 0.05 compared with the positive GM group; and ^¥^ = *p* < 0.05 compared with the GM + CH group.

**Figure 2:**
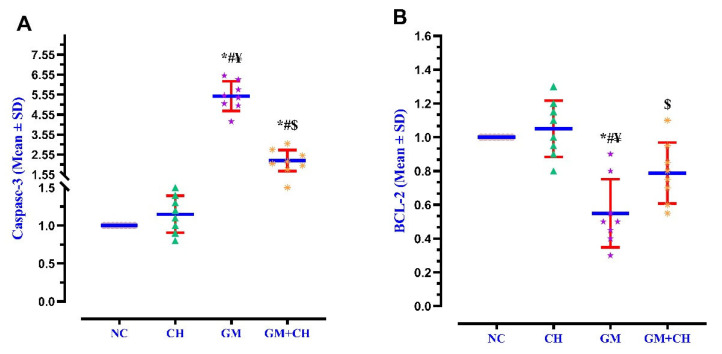
Interleaved scatter plots presenting the mean ± SD of the relative expression of Caspase-3 (**A**) and BCL-2 (**B**) in the renal tissues of the GM and GM + CH groups compared with their relative expression in the NC and CH groups. Dots, triangles, stars, and asterisks indicate the values of Caspase-3 and BCL-2 for the rats in each group (*n* = 8). Red error bars represent the ± standard deviation of the mean, and the blue lines indicate the mean values of the relative expression of these genes in each group. Statistical significance is indicated as follows: * = *p* < 0.05 compared with the NC group; ^#^ = *p* < 0.05 compared with the CH group; ^$^ = *p* < 0.05 compared with the positive GM group; and ^¥^ = *p* < 0.05 compared with the GM + CH group.

The expression of NF-κB, KIM-1AKT, Nrf2, Caspase-3, and BCL-2 in the renal tissues was compared across the study groups. The GM group displayed a significantly higher gene expression of NF-κB (5.2-fold), KIM-1 (4.8-fold), and Caspase-3 (>5.5-fold) compared to the NC group (Figure 1 and Figure 2), but the expression of AKT (0.43-fold), Nrf2 (0.51-fold), and BCL-2 (0.52-fold) was drastically lower than that of the NC group. In the CH + GM-treated group, the expression of NF-κβ, KIM-1, and Caspase-3 displayed a 2.67-, 2.05-, and 2.19-fold lower expression, respectively, compared to the GM group, while the expression of AKT, Nrf2, and BCL-2 displayed a 1.10-, 1.16-, and 0.75-fold higher expression, respectively, compared to the GM group and did not return to a normal level similar to that of the NC group (Figure 1 and Figure 2).

### 3.4. Renal Histology

**Figure 3:**
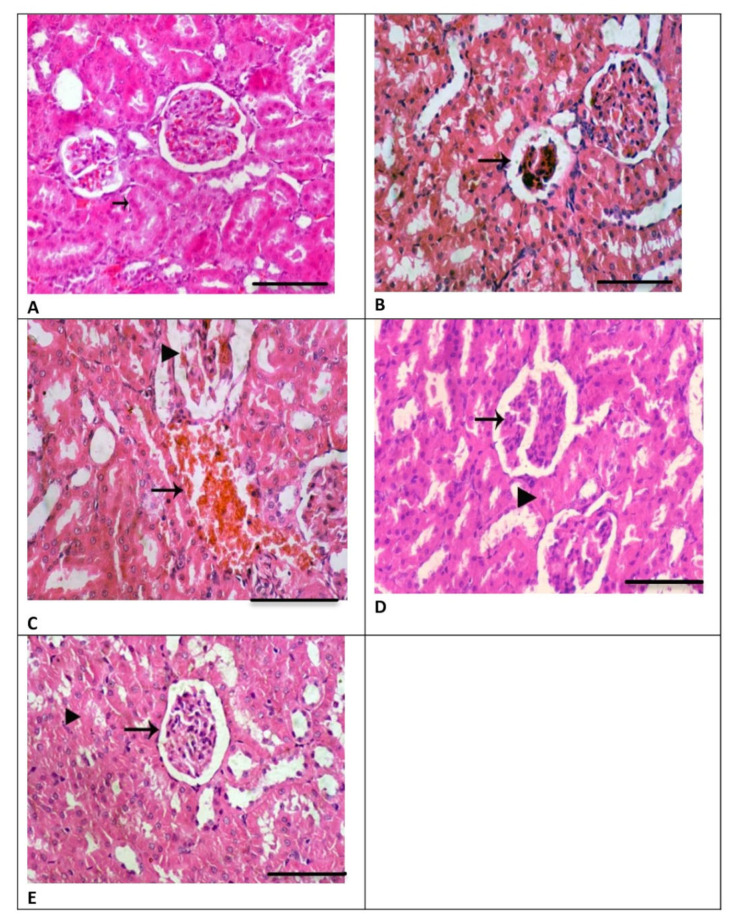
Renal histological features in all study groups, visualized via H&E staining (scale bar = 10 µm). (**A**) In the CH group, the kidney tissue shows normal renal glomeruli and renal tubules with a normal lining epithelium (as indicated by the arrow) (H&E, 400×). (**B**) In the GM-treated group, the kidney tissue shows the dissolution of renal glomeruli (as indicated by the arrow) (H&E, 400×). (**C**) In the GM-treated group, the kidney tissue also shows hemorrhaging in the interstitial tissue (as indicated by the arrow) that had replaced renal tubules and degenerative changes in the renal glomeruli (H&E, 400×). (**D**) In the GM + CH-treated group, the kidney tissue shows normal renal glomeruli (as indicated by the arrow) and cloudy swelling in the renal tubular epithelium (as indicated by the arrowhead). (**E**) In the GM + CH-treated group, the kidney tissue also shows scattered necrosis of the renal tubular epithelium (as indicated by the arrowhead) and normal renal glomeruli (as indicated by the arrow) (H&E, 400×).

**Figure 4:**
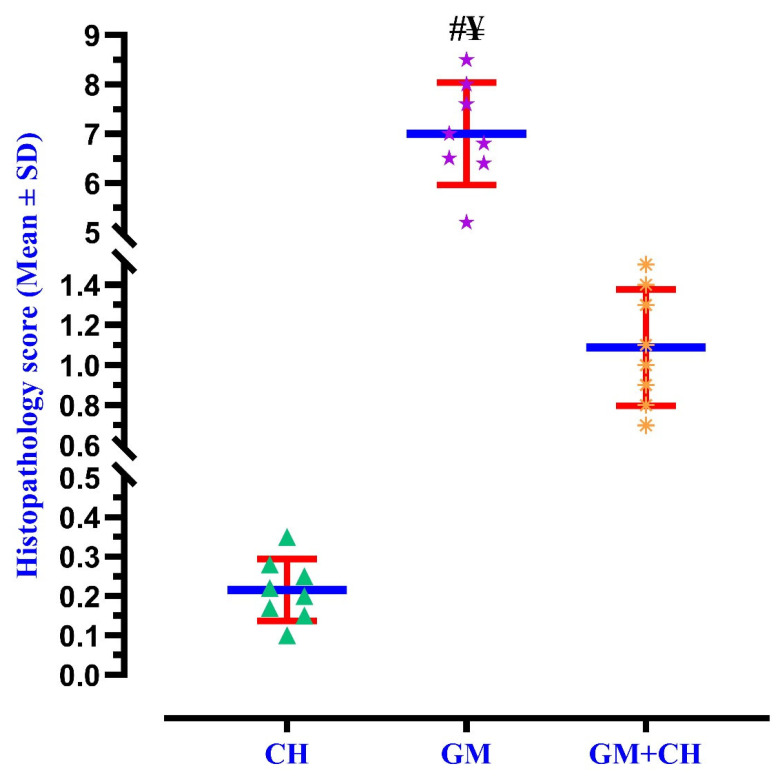
The histopathological damage scores in the renal tissues of all groups, displayed as bars in the graph (data are shown as mean ± SD). Triangles, stars, and asterisks represent the values of histological scores for each rat per group (*n* = 8), red error bars represent the ± standard deviation of the mean, and the blue line indicates the mean values of histological scores in each group. Statistical significance is indicated as follows: ^#^ = *p* < 0.05 compared with the CH group; and ^¥^ = *p* < 0.05 compared with the GM + CH group.

The renal tissues obtained from the CH group showed normal renal glomeruli and renal tubules with a normal lining epithelium (Figure 3A) and were like those of the NC group. However, in the GM-treated group, the renal tissue samples exhibited the disruption of Bowman’s capsule and dissolution of renal glomeruli, as well as a decrease in glomerular capillaries (Figure 3B). Moreover, there were scattered renal tubules and blood loss in the interstitial tissue that had replaced kidney tubules, along with degenerative changes in glomeruli (Figure 3C). However, in the CH + GM group, improved histological renal features comparable to those of the GM group were observed, as displayed in Figure 3D,E. The scoring analysis showed a significant difference in histopathological lesions in the GM group when compared to the CH and GM + CH groups (Figure 4).

## 4. Discussion

Treatment with GM can result in severe adverse effects such as nephrotoxicity and ototoxicity. GM-induced nephropathy is one of the most common causes of renal failure [34]. The present study found nephrotoxicity in the GM-treated group, as indicated by serum renal function tests showing increased levels of IL-1β, TNF-α, IL-18, and MDA in the kidney tissues, along with an amplified expression of NF-κB/KIM-1 and Caspase-3, decreases in antioxidant marker (GSH, CPx, CAT, and SOD) activities, and a decreased expression of the anti-inflammatory transcription factors Nrf2 and AKT. In the present study, a significant elevation in serum urea, creatinine, and urine protein was observed in the GM-treated rats compared with the control group, leading to severe histopathological damage to the renal tissues; these results are consistent with the findings reported by Laorodphun et al. [4]. GM-induced renal injury is associated with a substantial reduction in creatinine clearance and renal function capacity [4]. Acute exposure to GM results in its accumulation in renal tissues, producing progressive renal injury characterized by azotemia, polyuria, and proteinuria, along with the presence of various renal histopathological lesions, glomerulonephritis, and tubular degeneration [13,35,36]. Several authors have reported nephrotoxic effects and altered renal function test results after treatment with GM in pediatric and adult populations and experimental animals [34,37,38]. The most recent data demonstrate a sharp rise in the levels of ROS [39], proinflammatory cytokines, and apoptosis markers in renal tissues after GM exposure [3,14,38,40]. It is well established that GM toxicity can induce renal histopathological alterations and changes in lipid composition, causing renal macrophages to release proinflammatory cytokines such as IL1β and TNF-α [41]. The GM-treated group showed significant decreases in the GSH level and CAT, SOD, and GPx activities and a significant increase in the MDA level in the renal tissues compared with the NC group, indicating the presence of oxidative stress, as described in [13,42]. The oxidative effect of GM could be attributed to the accumulation of GM in renal tissues, resulting in a severe reduction in renal antioxidant enzyme activities [42]. ROS cause cellular damage through mitochondrial dysfunction, disrupt the electron transport chain, and subsequently reduce ATP production, in addition to causing cell membrane and DNA damage and reducing the capacity of the antioxidant system [3,43]. Renal oxidative damage induced by GM could be attributed to the augmented mitochondrial membrane permeability, followed by the release of cytochrome c, which initiates the activation of NF-kβ and, eventually, the activation of an apoptotic process [35,43,44]. In addition, GM enhances renal oxidative intracellular signaling by stimulating the expression of the proinflammatory cytokines KIM-1 and NF-κβ in renal tissues [10,13,36]. The transcription factors KIM-1 and NF-κβ play a crucial role in inflammation, apoptosis, and tissue damage [35,42,45,46]. Furthermore, an increase in GM in the renal tubules causes direct damage and enhances the expression of the inflammatory markers HSP-27 and NF-κB/KIM-1, while reducing the anti-inflammatory Nrf2/AKT pathways [1,14,47,48].

Only the CH-treated group showed insignificant changes in all the investigated parameters, including those associated with renal function, antioxidant system, inflammation, and histopathological features, compared with the control group. In a previous study, the LC50 of CH in rats was found to be 4350 mg/kg, supporting its prospective safety for prophylactic use against renal nephrotoxicity induced by inflammation and oxidative stress [49]. Additionally, previous studies have emphasized the non-toxic profile of CH, even at similar or higher doses taken over longer durations, in healthy animals and humans [50].

The co-treatment of CH with GM led to improvements in the renal function, blood urea level, creatinine level, creatinine clearance, urine volume, and urine flow, besides a reduction in the urine protein level, a lower apoptosis index, and preserved renal histology, when compared to the GM-treated rats. In addition, the co-treatment stimulated the renal antioxidant system (GSH, CPx, CAT, and SOD activities) and the expression of the anti-inflammatory marker IL10, as well as a reduction in oxidative markers, inflammatory cytokines (IL1β, TNF-α, and IL18), and inflammatory transcription factors (NF-κβ/KIM-1). Moreover, the rats treated with CH only showed comparable histological, biochemical, and apoptosis marker expression to those of the control rats.

CH has demonstrated promising protective effects against GM-induced nephrotoxicity by mitigating inflammation and oxidative stress via the hydroxyl and phenyl ring groups to neutralize free radicals, thereby blocking the oxidative process [51]. The co-treatment of CH with GM caused a significant elevation in GSH, CAT, SOD, and GPx activities, accompanied by a decrease in MDA, when compared with the GM-treated rats; these results are in agreement with those reported by Mani and Natesan [19]. In the present study, the co-treatment of CH with GM exhibited a reno-protective effect, with a significant reduction in creatinine and urea levels, reduced proteinuria, and improved creatinine clearance to near the control level, thereby adjusting the glomerular filtration rate through the anti-inflammatory and antioxidant properties of CH [19,26]. Numerous studies have demonstrated the antioxidant, anti-inflammatory, and mitochondrial protective effects of CH using various nephrotoxic models [26,48,52]. In this study, the group treated with both CH and GM showed significant histological improvement. Numerous studies have shown the reno-protective effect of CH against nephrotoxic agents by reducing renal damage and the expression of apoptosis markers [26]. A previous study demonstrated that CH protects against renal tubular damage by reducing the levels of the proinflammatory cytokines IL1β and IL6, as well as those of apoptosis markers, in a renal ischemia model [23]. In the same aspect, CH has a protective effect against testicular injury induced by lead toxicity by stimulating the expression of antioxidant and anti-inflammatory markers, as well as anti-apoptotic pathways [52].

To understand the molecular mechanisms underlying the antioxidant and anti-inflammatory effects of CH against GM-induced oxidative injury, the expression of the transcription markers Nrf2/AKT and NF-κB/KIM-1 was investigated. Interestingly, the transcription factors Nrf2 and AKT could stimulate mitochondrial functions and augment the antioxidant system in diverse kidney illnesses, significantly reducing oxidative markers, proinflammatory cytokines, and inflammatory transcription markers such as NF-κβ/KIM-1 [14,17,26,52,53]. Moreover, AKT has been reported to downregulate TGF-β and apoptosis pathways in renal tissues in experimental models, thereby attenuating nephrotoxicity and renal damage [54,55]. On the same line, CH ameliorates cardiac dysfunction by stimulating the expression of the transcription factors Nrf2 and AKT [56,57]. The therapeutic importance of CH has been documented for neurological disorders [58], gastric cancers, pancreatic and lung adenocarcinoma [59], cardiovascular illnesses [22], and hepatorenal diseases [21,25].

## 5. Conclusions

The present study found that oxidative stress and apoptotic response play a crucial role in GM-induced nephrotoxicity. The co-treatment of CH with GM alleviates the renal toxicity induced by GM by improving the antioxidant system, attenuating renal oxidative damage and inflammatory response, and improving renal apoptosis. The protective effect of CH was found to occur through its modulation of the levels of antioxidant markers, the anti-inflammatory Nrf2/AKT and NF-κB/KIM-1 pathways, and anti-apoptotic activities. Future comparative studies are warranted to evaluate the therapeutic efficacy of CH against other known natural antioxidants and/or pharmacological therapies in the treatment of nephropathy induced by nephrotoxic drugs.

## Figures and Tables

**Table 1:** Sequences of the primers and probes used for RT-PCR.

Protein-Encoding Gene	5′-3′ Sequence	
	Forward	Reverse
AKT	5′-TCT ATG GCG CTG AGA TTG TG-3′	5′-CTT AAT GTG CCC GTC CTT GT-3′
Nrf2	5′GAG GAU GGG AAA CCU UAC UTT-3′	5’AGU AAG GUU UCC CAU CCU CTT-3′
NF-κB	5′-AAG CAC TCG GAT ACA GCA GC-3′	5′-AGT CGT CAT AGG GCA GCT CA-3′
KIM-1	5′TTC AGG AAG CTG AGC AAA CAT-3′	5′CCC CAA CAT GTC GTT GTG ATT-3′
Caspase-3	AGTTGGACCCACCTTGTGAG	AGTCTGCAGCTCCTCCACAT
BCL-2	CACCCCTGGCATCTTCTCCTT	AGCGTCTTCAGAGACAGCCAG
Actin	5′-CAC GAT GGA GGG GCC GGA CTC ATC-3′	5′-TAA AGA CCT CTA TGC CAA CAC AGT-3′

**Table 2:** Histopathological semi-quantitative analysis.

Score	Tubular Damage	Glomerular Changes	Vascular Changes
0	None	None	None
1	Mild tubular degeneration or necrosis	Mild glomerular shrinkage with minimal increase in uriniferous space	Mild hemorrhage
2	Moderate tubular degeneration or necrosis	Moderate glomerular shrinkage with mild increase in uriniferous space	Moderate interstitial hemorrhage
3	Severe tubular necrosis	Severe glomerular shrinkage with high uriniferous space	Severe interstitial hemorrhage

**Table 3:** Blood and 24 h urine values (mean ± SD) of renal biochemical function in the study groups.

		NC Group	CH Group	GM Group	CH + GM Group
Serum	Creatinine (mg/dL)	0.32 ± 0.10	0.37 ± 0.08	0.63 ± 0.15 *^#¥^	0.42 ± 0.09 ^$^
	Urea (mg/dL)	35.62 ± 5.3	34.87 ± 5.1	61.9 ± 10.1 *^#¥^	39.62 ± 6.4 ^$^
	Total protein (g/dL)	7.05 ± 0.89	7.43 ± 0.92	5.91 ± 0.22 *^#¥^	6.90 ± 0.78
	Albumin (g/dL)	4.37 ± 0.52	4.55 ± 0.36	3.57 ± 0.52 *^#¥^	4.16 ± 0.44
24 h Urine	Urine volume (mL)	8.45 ± 0.88	8.50 ± 1.01	5.72 ± 0.48 *^#¥^	7.47 ± 0.90 ^$^
	Urine flow (µL/min)	5.86 ± 0.61	5.90 ± 070	3.97 ± 0.34 *^#¥^	5.19 ± 0.63
	Creatinine (mg/dL)	41.1 ± 6.02	42.5 ± 5.55	25.4 ± 3.20 *^#¥^	38.3 ± 2.50 ^$^
	Creatinine clearance (mL/min)	0.55 ± 0.13	0.49 ± 0.11	0.12 ± 0.05 *^#¥^	0.35 ± 0.10 *^#$^
	Total protein (mg/dL)	3.80 ± 0.39	3.97 ± 0.50	11.71 ± 1.98 *^#¥^	6.41 ± 1.06 *^#$^

Note: NC = negative control group, CH = chrysin-treated group, GM = positive gentamicin control group, and CH + GM = positive gentamicin + chrysin group; * = *p* < 0.05 compared with the NC group, ^#^ = *p* < 0.05 compared with the chrysin-treated group, ^$^ = *p* < 0.05 compared with the positive gentamicin group, and ^¥^ = *p* < 0.05 compared with the positive gentamycin + chrysin group.

**Table 4:** Renal tissue concentrations (mean ± SD) of proinflammatory and oxidative stress markers in the study groups.

	NC Group	CH Group	GM Group	CH + GM Group
TNF-α (pg/mL)	58.1 ± 6.72	56.1 ± 5.42	190.1 ± 13.61 *^#¥^	109.1 ± 18.18 *^#$^
IL1β (pg/mL)	37.6 ± 13.11	33.1 ± 6.64	187.2 ± 17.90 *^#¥^	89.2 ± 13.12 *^#$^
IL6 (pg/mL)	63.1 ± 7.37	62.8 ± 8.01	248.8 ± 20.21 *^#¥^	103.6 ± 18.78 *^#$^
IL18 (pg/mL)	13.6 ± 3.11	12.50 ± 2.92	47.25 ± 6.71 *^#¥^	32.01 ± 4.78 *^#$^
IL10 (pg/mL)	37.6 ± 7.63	39.1 ± 4.92	19.8 ± 4.05 *^#¥^	35.1 ± 5.28
HSP25 (ng/mL)	1.73 ± 0.66	1.80 ± 0.40	5.98 ± 1.07 *^#¥^	2.15 ± 0.74
GSH (mg/g)	33.8 ± 5.44	34.4 ± 5.15	20.2 ± 5.07 *^#¥^	29.6 ± 5.26
SOD (U/g)	50.1 ± 6.68	52.6 ± 6.16	28.5 ± 5.47 *^#¥^	46.5 ± 5.70
GPx1 (µg/mg)	5.2 ± 0.1.1	5.5 ± 0.73	3.1 ± 0.74 *^#¥^	4.5 ± 0.44 ^#^
CAT (U/mg)	236 ± 29.8	239 ± 35.3	170 ± 18.70 *^#¥^	223 ± 22.6
MDA (nmol/g)	26.7 ± 7.12	25.6 ± 5.09	55.6 ± 7.85 *^#¥^	31.1 ± 7.60
H_2_O_2_ (μM/g)	1.70 ± 0.34	1.62 ± 0.29	9.5 ± 1.17 *^#¥^	3.1 ± 0.76 *^#^
Protein Carbonyl (nmol/g)	0.56 ± 0.20	0.53 ± 0.10	5.4 ± 0.92 *^#¥^	0.73 ± 0.13

Note: NC = negative control group, CH = chrysin-treated group, GM = positive gentamicin control group, and CH + GM = positive gentamicin + chrysin group; * = *p* < 0.05 compared with the NC group, ^#^ = *p* < 0.05 compared with the chrysin-treated group, ^$^ = *p*< 0.05 compared with the positive gentamicin group, and ^¥^ = *p* < 0.05 compared with the positive gentamycin + chrysin group.

## Data Availability

The data supporting the findings are not publicly available due to privacy or ethical restrictions. The data are available upon reasonable request from the corresponding author.
